# Patient and befriender experiences of participating in a befriending programme for adults with psychosis: a qualitative study

**DOI:** 10.1186/s12888-020-02776-w

**Published:** 2020-07-14

**Authors:** Erin Burn, Agnes Chevalier, Monica Leverton, Stefan Priebe

**Affiliations:** 1 Unit for Social and Community Psychiatry, (WHO Collaborating Centre for Mental Health Service Development), Queen Mary University of London, London, UK; 2 East London NHS Foundation Trust, London, UK; 3 Division of Psychiatry, University College London, London, UK

**Keywords:** Volunteering, Befriending, Psychosis, Experiences

## Abstract

**Background:**

Befriending is a popular form of volunteering in healthcare, and research suggests that it can be beneficial for people with mental illness. This study aimed to explore the experiences of a large sample of volunteer befrienders and patients who participated in the VOLUME trial, testing the efficacy of a structured befriending programme for individuals with psychosis. This is the first study to explore the specific challenges and benefits of befriending in both volunteers and patients in this population within the same programme.

**Methods:**

A series of in-depth semi-structured interviews were conducted with 34 volunteer befrienders and 28 participating patients. All participants who had taken part in at least one befriending session were invited to be interviewed about their experiences with the aim of including a wide range of views, including those who were more or less engaged with the befriending programme. The data were analysed using Thematic Analysis.

**Results:**

Four broad themes were developed from the analysis of the befriender and patient interviews which, although were largely discrete, captured the overall experiences of participating in the befriending programme: 1) Bridging the gap, 2) A genuine relationship that developed over time, 3) A big commitment, and 4) A flexible approach.

**Conclusions:**

These results further support that, befriending programmes for individuals with psychosis can be a worthwhile experience for both befrienders and patients. However, participation also requires perseverance and flexibility from both sides. Different factors, such as incorporating participant preferences for frequency of meetings, have to be considered in the development and management of a befriending programme in order to provide effective support to both befrienders and patients.

## Background

Befriending is a common form of volunteering generally involving supportive one-to-one companionship with a non-professional over a regular period of time [1, 2]. Commonly, the focus of the befriending relationship is for the befriender and befriendee to engage in meaningful social and leisure activities [3, 4]. Befriending has been shown to have benefits for people with both mental and physical illnesses, such as a reduction in symptoms [5], social isolation [6, 7], and improved patient reported outcomes, including well-being and quality of life [8]. Befriending can be distinguished from peer support interventions that also aim to provide support and promote social inclusion, but have differential mechanisms in bringing about this change. As those in peer support initiatives have a shared lived experience of mental illness, the relationship incorporates a greater focus on mutual support and self-recovery [9], and thus provides different support functions to befriending [1, 8].

Previous research has predominantly focused on assessing the benefits of befriending rather than exploring the experiences of participating in these programmes. Even when these experiences were considered, it was largely from the befrienders’ viewpoints [10]. A systematic review by Hallett and colleagues [4] and updated by Toner and colleagues [11] synthesised the experience of befrienders, who were participating in several heterogenous befriending programmes for individuals with severe mental illness. Despite the programmes differing in terms of structure, including the duration of the programme and the level of training and supervision offered, befrienders reported a largely positive experience of befriending. Research exploring both the perspectives of befrienders and patients is limited to a handful of studies, the majority of which include data collated from multiple befriending programmes. These studies, however, indicate that befriending is predominantly a positive experience for both participant groups, but state that there are some challenges, including confusion about the role of a befriender and the level of commitment required [3, 10, 12].

The benefits of befriending is thought to be particularly relevant for individuals with psychosis, who experience higher levels of social isolation than the general population [13, 14]. Recent research has aimed to establish the effectiveness of befriending in this population, and which showed positive gains in social outcomes (increased social contacts) despite variable implementation of the programme. However, no significant difference was found between the control and intervention group in subjective quality of life, self-esteem or symptoms [7]. To our knowledge, there has been no research that has explored the specific experiences of participating in befriending programmes for people living with psychosis.

The present study therefore aims to explore the experiences of a large sample of both befrienders and patients who participated within the same structured befriending programme for individuals with psychosis.

## Methods

### Sample

Interviewees were recruited from a larger sample of people participating in the VOLUME trial, a randomised controlled trial testing the efficacy of a befriending intervention for people diagnosed with a psychotic disorder conducted across ten community services in East London [7]. The full protocol and findings of this trial has been published elsewhere [7, 15]. It is worthy of note, that the primary aim of the current study is to report on the experiences of a large sample of participants who took part in the same structured befriending programme, rather than a process evaluation of the VOLUME trial per se. Briefly, befrienders and patients were matched due to preference (e.g. on gender and location) and availability. Befriending pairs were encouraged to meet for 1 h on a weekly basis, over the period of 1 year, and the focus was on encouraging the patient to engage in more activities outside of the home. To do this, befriending pairs were given an activity booklet detailing inexpensive activities in the local area, and during the year patients and befrienders were invited to monthly socials (such as ice skating, picnics, etc.) as an opportunity to meet and interact as a group. Once the programme was formally finished (i.e., after 1 year), patients and befrienders could continue the relationship if both chose to, although support from the programme was discontinued.

Patients were eligible to participate in the programme if they were between 18 and 65 years of age, had a diagnosis of schizophrenia or a related disorder (ICD: 10 F20–29), had capacity to provide written consent, and had sufficient command of the English language to participate in the programme. Befrienders were eligible to participate if they were over 18 years of age, had sufficient command of English, had no criminal record, and were not currently receiving secondary mental healthcare (to distinguish befriending from peer support).

Attempts were made by telephone to contact all participants who had taken part in at least one befriending session and invite them to an ending interview. This ensured a varied range of demographic characteristics and included both participants who were more or less engaged with the befriending programme. Ethical approval was received from Camden and Kings Cross Research Ethics Committee (15/LO0674). Written informed consent was obtained for the VOLUME trial, which also included this interview study.

### Procedures

The semi-structured interview schedule was developed in collaboration with a panel formed of people with lived experience, who also contributed to the development of the befriending programme. The interview schedule was designed to be open-ended and in-depth with the participants guiding the direction of the interview, but included key topics aimed at structuring the conversation. Topics included: motivations for participating, the relationship between the befriender and patient and how this might have changed over the course of year, the impact of participating in the befriending programme, as well as what worked well and any challenges that were faced.

Participants were contacted and invited to an ending interview by telephone. Interviews were conducted by four female graduate researchers in a variety of locations based on participant preference, including community mental health premises or the participants’ own homes if a quiet and private space was available. Participants were compensated with £15 for being interviewed. As part of the strategy to disseminate results, participants were invited to an event to hear about the findings from this study.

### Data analysis

Interviews were audio-recorded, transcribed verbatim by an external transcription company, and all transcripts were checked by the research team to ensure accuracy. Anonymised transcripts were uploaded to NVivo version 11 and inductive Thematic Analysis, which followed the guidelines outlined by Braun and Clarke [16], was selected in order to best explore the views and experiences of participants across a large dataset. All analysts were female psychology post-graduates and researchers trained in qualitative analysis.

Although the befrienders’ and patients’ experiences of the programme were analysed separately to account for the variation in the different roles and experiences that both were likely to have had, the same protocol was followed. Firstly, all analysts read the full set of transcripts to familiarise themselves with the data. Ten transcripts from each set of interviews were then selected on the basis of being the most representative of the larger data-set as outlined at the familiarisation stage, for line-by-line analysis. This was completed independently by two analysts (patient analysis: ML & AC; befriender analysis: EB & AC).

In the second stage of analysis, the analysts developed a coding framework that was based on discussion surrounding the initial codes and included resolution of any discrepancies and agreement on how the codes may be interlinked. More specifically, all initial codes that were generated by the two analysts were written on separate post-it notes, and were then grouped based on potential patterns that emerged, indicating preliminary themes and sub-themes.

The coding framework was then applied to the remaining patient and befriender transcripts by the lead analysts, ML and EB respectively. During this process, any new codes that were not captured by the original framework were discussed with the larger research team iteratively, and where necessary, the coding framework was modified. In the final stage of the analysis, the analysts examined how the codes could be interlinked and modified to develop the overarching themes of the data.

## Results

In total, 34 (66%) of 51 befrienders and 28 (57%) of 49 patients who had participated in at least one befriending session, agreed to an interview at the end of the 12-month intervention. The participants interviewed varied considerably in the number of befriending sessions attended; patients attended 1–42 sessions, with a mean of 17.5 sessions attended, and befrienders attended 1–42 sessions, with a mean of 18 sessions attended). The remaining befrienders and patients either declined to be interviewed (e.g., did not want to be audio-recorded) or were lost to follow-up (e.g., were not able to be contacted post-intervention). Interviews ranged in length from 20 min to 2 h. Demographics of the participants are presented in Table 1. In contrast to their paired patients, befrienders were mostly female, younger, and more likely to be in paid employment.

**Table 1:** Socio-demographic characteristics of patients and befrienders

Patient characteristics (*n* = 28)	
Gender (n, %)	
Female	12 (42.9%)
Male	16 (57.1%)
Age (Mean, SD)	43 (10.3)
Ethnicity (n, %)	
White	7 (25%)
Black / Black British – African	6 (21.4%)
Black / Black British – Caribbean	5 (17.9%)
Bangladeshi	5 (17.9%)
Other	5 (17.9%)
Years since diagnosis (Mean, SD)	25.52 (11.30)
Employment status (n, %)	
Paid employment	1 (3.6%)
Full time education or training	1 (3.6%)
Retired	1 (3.6%)
Unemployed	25 (89.3%)
Withdrew from the scheme (n, %)	4 (14.3%)
**Befriender characteristics (*n*** **= 34)**	
Gender (n, %):	
Female	24 (70.6%)
Male	10 (29.4%)
Age (Mean, SD)	29.24 (9.95)
Ethnicity (n, %)	
White	17 (50%)
Black / Black British – African	6 (17.6%)
Black / Black British – Caribbean	1 (2.9%)
Bangladeshi	2 (5.9%)
Indian	1 (2.9%)
Pakistani	1 (2.9%)
Other	6 (17.6%)
Employment status (n, %)	
Full time employment	12 (35.3%)
Part time employment	8 (23.5%)
Full time student	4 (11.8%)
Unemployed	4 (11.8%)
Retired	1 (2.9%)
Other	3 (8.8%)
Did not disclose	2 (5.9%)
Previous experience of volunteering (n, %)	
Yes	24 (70.6%)
No	10 (29.4%)
Withdrew from the scheme (n, %)	6 (17.6%)

Four overarching themes were developed from the data and are presented along with their sub-themes in Table 2. These were: 1) Bridging the gap, 2) A genuine relationship that developed over time, 3) A big commitment, and 4) A flexible approach.

**Table 2:** Themes

Main Theme	Sub-themes ***patient***	Sub-themes ***befriender***
**Bridging the gap**	Normalising schizophrenia	Changing perceptions of schizophrenia
	Is the gap too big to bridge?	My match was an easy one
**A genuine relationship that developed over time**	Developing a genuine and reciprocal friendship	Attuning to my match
		Befriending can be emotional
**A big commitment**	It doesn’t feel like they have time for me	Befriending is a bigger commitment than I originally thought
	Befriending is a voluntary role	It’s volunteering so there is no obligation
**A flexible approach**	The scheme needs to be flexible to differing needs	The balance between pushing and patronising
	Achieving goals vs. having someone to talk to	

### Bridging the gap

Some patients expressed feeling isolated from society due to having psychosis. As members of the community, befrienders were viewed by patients as being in a good position to bridge this perceived gap between mental illness and society. For some, this was simply the notion that the befriender, as a so called ‘normal’ member of society wanted to spend time with them.

*“That’s what was helpful, being treated like I was normal and worthy of respect by a member of society who was considered to be a normal respectable member of society. In that respect, that makes you feel you fit in, you know.” (Patient 131, attended 12 meetings)*

For some patients, however, the differences between the befriender and themselves were felt to be too large, with some feeling judged by their befriender.

*“She was arrogant. […] because she was at college and that, because I never went to college, she asked about my college and school, and started asking all personal questions. I don't like that.” (Patient 15, attended 13 meetings)*

Many befrienders admitted to being initially influenced by false preconceptions of psychosis, common throughout society and depictions in the media. This in turn affected how nervous they were when joining the scheme. As a result of taking part in the befriending programme, many befrienders reported that their perspective changed and they developed a better understanding of the reality of psychosis.

*“My mother thinks that schizophrenic people go around killing people on the streets of London and that’s what most people think and that’s what potentially I may have had a slight thought about before doing this scheme and then meeting someone like [patient] and realising that she isn’t harmful to no one” (Befriender 23, attended 42 meetings)*

Nevertheless, this changed perception of psychosis was not always generalised beyond their own befriending match. Some befrienders felt that they had been lucky to befriend an ‘easy match’, and that other patients in the programme were more challenging, expressing behaviours more in line with their initial perceptions of psychosis.

*“To be able to see especially for those social events because there were people different from [patient] and [patient]’s a mild situation. You can’t differentiate her from anyone else. She looks completely normal. And I was fortunate” (Befriender 53, attended 22 meetings)*

### A genuine relationship that developed over time

To make a new friend and find companionship was one of the key motivators for many patients to join the scheme as they often expressed feeling lonely and isolated with few, if any, social contacts. For many this was achieved, with patients feeling that the befriending relationship developed into a friendship over the course of the programme.

*“Because even for me I really value the friendship that me and her have made now, It’s always nice to make new friends, so I think having her in my life now, I do see us being friends for a long time” (Patient 103, attended 7 meetings)*

Befrienders and patients spoke about how having certain commonalities, such as cultural background and mutual interests, enabled a friendship to develop between them. For some, the befriender opening up about their own life or even discussing their own difficulties also aided the development of a real friendship with reciprocal support.

*“I do discuss my personal issues with her. She discuss[es] her own personal issues with me and she does ask me where I’m needing advice for some certain things she does advise me. And when she did advise, I did advise her, we became close.” (Patient 17, attended 23 meetings)*

Many befrienders stated that as the relationship developed over time, they could attune to their match, which enabled them to pick up on subtle signals to understand how they were feeling.

*“Like if there was a really busy place that we’d been to, like I say, I could see the change in her body language and needing to just leave. So, when we had the social where it was at a football stadium it was really busy and as we got out of the station, because it was really loads of people, I could tell she was like shutting down a bit” (Befriender 31, attended 29 meetings)*

Whilst being able to relate to their match was viewed as important, with many befrienders reflecting that empathy was vital for the role, this also had a downside in that they could become too emotionally invested.

*“I think one of the reasons I could do it and I was quite good I think at befriending him, is about empathy and emotional availability but I think if you have those things it can also be quite painful and quite intense and quite emotionally distressing” – (Befriender 39, attended 19 meetings)*

This became especially poignant towards the end of the scheme where several befrienders felt that terminating the relationship could be cruel for the match who had become used to receiving social support from a befriender.

*“I think that it’s quite cruel that it cuts off after a year and you then perhaps don’t have any contact with the person again. I do feel it’s quite harsh, that they have somebody once a week every week and then they have a social once a month and then that’s gone” (Befriender 60, attended 38 meetings)*

### A big commitment

Participation in the programme often felt like a big commitment to befrienders, and often a larger commitment than was originally expected. This was often due to unforeseen lifestyle changes for the befriender, competing commitments, and the additional impact of travelling to and from befriending appointments, particularly when the befriendee did not live close by.

*“I had to try and coordinate having my own life, my family and I’ve got a boyfriend [...], so it did get at points some weekends where I was thinking, I’d love to spend it with my boyfriend or I’ve love to go away with my friends but I haven’t seen [patient] for a week and I need to see her” (Befriender 23, attended 42 meetings).*

Many befrienders felt unable to meet their match on a weekly basis, which sometimes led to infrequent meetings, or having to cancel appointments last minute due to competing demands.

*“I think as the year progressed because I was becoming a little bit more busy and I was finding the commitment a bit hard but I didn’t kind of want to let go of the commitment altogether especially because we’d built a good bond” (Befriender 62, attended 6 meetings).*

However, some befrienders felt that as the programme was voluntary there was not the same obligation to be consistent within the role as there would be for a paid job. Some spoke about being attracted to this voluntary role in particular, because of the flexibility, and freedom to negotiate the timing and location of meetings.

*“Like obviously it’s volunteering, so it’s nothing that you have to do every week, it’s something if you can make time and if your match has time then it’s nice to meet up” (Befriender 35, attended 14 meetings).*

Patients had different views on the level of commitment they expected from their befriender. Some felt that the befriender could not provide the time that they felt they ought to give, whilst others expressed awareness that their befriender was a volunteer, and therefore understood that they had other commitments and priorities.

*“She works ‘n all and she hasn’t got time for me most of the time […] she doesn’t have time for me and I don’t have time for her” (Patient 45, attended 13 meetings).*

*“She was a student, so she couldn't always give me time. Plus she had exams, so I kind of understand she couldn't always make time for me. But when she did make time, it was really enjoyable.” (Patient 46, attended 21 meetings).*

### A flexible approach

Both volunteers and patients agreed that the roles of the befriender and the programme needed to be flexible in acknowledging that every person is individual in terms of their needs. Patients appreciated a flexible approach to befriending, where they could be supported to achieve their goals, or to just have someone there to talk to.

*“Each day is different; each day is a different battle. On a good day I will challenge myself. On a bad day I will hibernate, sleep.” (Patient 94, attended 25 meetings)*

*“You might look at an activity as a befriender, yes, somebody to do things with, but sometimes people are so stressed they just need somebody to talk to.” (Patient 30, attended 11 meetings)*

Some befrienders were mindful of the need to encourage their match without appearing to be patronising; therefore, finding a balance in achieving this was necessary, but the difficulty of attaining this balance was acknowledged. Other times it felt necessary to gently push their match outside of their comfort zone and that this in hindsight was appreciated.

*“There’s a feeling for me of I feel like it’s really delicate not to be the apparently well person coming into help the unwell person do things they should really be doing. I really didn’t want to do that. […] I didn’t want to be pushing him out of his life” (Befriender 39, attended 19 meetings)*

*When we first met, he’d be like […] “I’m feeling really tired today,” or, “I’m feeling really unwell,” and then at the end when we were leaving […] he’d just say, “I’m really glad I came out today, it did sort of help.” (Befriender 33, attended 15 meetings)*

## Discussion

Four broad themes largely capture the experiences of both volunteers and patients who participated in the same befriending programme for individuals with psychosis. The first theme, ‘Bridging the gap’, indicates that befriending can be a means of normalising psychosis within society. For patients, it was empowering to have access to a non-judgemental and supportive segment of society, and the volunteers noted that through exposure to someone with psychosis they became aware of any misconceptions they may have held prior to volunteering. However, this change in attitude may not have always extended beyond the befriending pair, as the befriender’s change in attitude appeared confined to their matched patient, as opposed to being generalised to other people experiencing psychosis. The second theme, ‘A genuine relationship that developed over time’, explores the relationship that developed between the befriending pairs over the one-year period that they were participating in the scheme. Many of the participants felt that a genuine and authentic relationship had developed between them. Some befrienders, however, reflected that through developing a relationship with their match they became too emotionally invested. In the third theme, ‘A big commitment’, the befrienders highlighted the reality that befriending can be a big commitment, and that balancing regular meetings with the patient and other commitments, such as work, family, and social life, could be challenging in a voluntary role. Befrienders differed on the level of commitment that they were willing to invest, but the general consensus was that meeting once per week was too much. Similarly, patients differed on the level of commitment that they expected from their befrienders. In the final theme, ‘A flexible approach’, participants iterated that the befriending programme needs to be flexible, and not too rigid in its approach to support the differing needs and goals of the participants. It was felt that the focus of the programme therefore should not only be to encourage the patients to engage in activities, but should also provide them with someone to talk to. Befrienders reported that they sometimes struggled with this balance, and were unaware of the extent to which it was appropriate to push their match.

### Comparison with the literature

The benefits and challenges of befriending people with psychosis appear to be non-specific to the benefits and challenges of befriending individuals with mental illnesses in general. The findings from this study are consistent with previous literature indicating that befriending programmes for people with mental illness can provide numerous benefits for both the befrienders and patients, but concurrently can also be challenging [3, 4, 10, 12].

A benefit of befriending that was explored within the present study suggests that befriending might be a means of challenging society’s prevailing negative perceptions towards mental illness due to increased exposure and familiarity. A study by Toner and colleagues [17] found that befrienders had less desire for social distance than the general population, however, a degree of social distance still remains. Interestingly in this present study, the befrienders change in attitude towards psychosis was not always generalised past their own match, which could suggest some desire for social distance still remains.

Many of the challenges that were identified in this study, such as the level of commitment required to participate, were not insurmountable to the overall success of the programme. Furthermore, many of the challenges that were experienced could be addressed through flexibility on the part of the befriender and the programme. It has been suggested that there is not a one-size-fits-all approach to befriending individuals with mental illness, as there is a limited consensus on a range of befriending options including the variability and longevity of the programme, as well as the type of the relationship that is developed with the befriender [10, 18].

Similarly, being too rigid in the objectives of the befriending programme can create confusion over the role of the befriender. In a review of how befriending has been conceptualised in previous literature, Thompson and colleagues [1] found that there was a spectrum of different befriending practices. On one side of the spectrum, befriending was conceptualised as more of a professional relationship, which is characterised as being boundaried and goal-focused. On the other side, befriending has been likened to the development of a natural friendship, which in turn has been linked to a greater risk of emotional turmoil [1]. Both the befrienders and patients in this present study largely conceptualised the role of a befriender as closer to a genuine friend as opposed to a more professional relationship. This could have caused some role ambiguity for the befriender, who may have felt pulled in different directions by the needs of the patient and the requirements of the programme, which were goal-focused in encouraging the patients to engage in more activities. Toner and colleagues [18] found that the majority of patients who were surveyed about their preferences for a befriending programme, favoured having someone to listen to and support them, rather than pushing them to do more activities. This finding might reflect some of the discomfort that befrienders felt in trying to push their match to achieve specific goals, which was sometimes met with resistance.

### Strengths and limitations

To our knowledge, this is the first study to evaluate both patients’ and befrienders’ experiences of participating in a structured befriending programme specifically designed for individuals with psychosis. Therefore, these findings enrich the understanding of the factors that may affect the uptake and acceptability of befriending programmes in this population. This study may be helpful in the design of future programmes to optimise acceptability.

A major strength of this study is the large sample size and, to date, this is the largest known study exploring both the experiences of volunteers and patients who have participated in befriending programmes for individuals with mental illness [3, 10, 12]. A second advantage of this study is that all participants took part in the same structured befriending programme, and therefore the participants’ perspectives were not affected by variation in the structure or design of different befriending programmes. Furthermore, we used a broad sampling strategy by inviting every participant who had took part in at least one befriending session to be interviewed. This ensured that we got a varied range of opinions and experiences, and included those who were more or less engaged with the programme.

There were several limitations to this study: firstly, participants were recruited only from community services across three East London boroughs (Newham, Tower Hamlets, and City & Hackney). These boroughs are in an urban and multicultural area marked by specific challenges, including a high deprivation index and a transient population. It is therefore unknown whether the accounts provided by the volunteers and patients in this study would be reflective of befriending programmes conducted in different locations, including semi-urban and rural locales.

Additionally, despite all participants coming from the same befriending programme, there was large variation in how often they met with their match (between 1 and 42 meetings). Participants who accepted an ending interview also tended in general to have been more engaged with the intervention than those who declined to be interviewed or were lost to follow up. The experiences of the participants who met with their match less frequently were likely to vary substantially from those who met with their match more often, as many of the benefits that were discussed within this study appeared to become more apparent over time. Furthermore, the sample of befrienders who participated in this study had different characteristics than found in previous befriending studies; most notably they were, on average, substantially younger than befrienders reported elsewhere [11, 12, 17]. This also meant that many of the befrienders in this study were younger than the patients they were matched with. It is not improbable that some of the challenges and benefits that were discussed by participants in this study may be specific to the different demographic of befrienders observed in this study.

Despite the themes being developed inductively from the content of the transcripts, it is likely that due to the analysts’ backgrounds in mental health research, the interpretation of the data was to some extent shaped by this previous experience. Furthermore, the second and third authors were researchers on the larger VOLUME trial which may have led towards biases in placing more emphasis on the more positive experiences of participating in the intervention.

### Implications

Befriending has shown to be a valuable resource in the care of individuals living with mental illness, and this study indicates that the benefits and challenges of befriending individuals with psychosis are similar to befriending individuals with mental illness in general. Understanding the experiences of befrienders and patients has implications for the design and implementation of future programmes to fully optimise befriending as a resource. In particular, additional research is needed to specifically explore reasons for ‘drop-out’ from befriending programmes and to identify factors that affect engagement. Preliminary analysis from this study suggests that befriending programmes that are too rigid in their design may be unhelpful. This seems particularly pertinent when befriending programmes are too prescriptive in their aim, for example, the focus is solely on achieving goals as opposed to just providing a befriender as someone for the patient to talk to. The development and implementation of more flexible programmes would allow for participant preference to be accommodated, particularly in the type of befriending relationship that is formed, as well as the longevity and variability of the programme. Furthermore, there are also implications for the training and supervision of befrienders, which must acknowledge that befriending can be demanding and emotional. Both training and ongoing support should be offered for a range of issues faced by befrienders, including role ambiguity and managing relationships ending.

## Conclusion

Befriending is a valuable resource in mental healthcare that has been shown to be beneficial for both patients and befrienders; at the same time, befriending can also be challenging and a flexible approach is required. Taking into account participant preference in both the design of befriending programmes and in the support provided to volunteers and patients during participation in a programme may maximise the benefits that befriending can be provide. Befriending is just one aspect of a growing area of person-centred mental healthcare that includes other interventions such as peer support. The findings on befriending reported in this study likely share common challenges and benefits with these other interventions, and comparisons between them should be investigated in future research.

## Data Availability

The datasets used and/or analysed during the current study are available from the corresponding author upon reasonable request.
